# Is functional cure achievable in children perinatally exposed to HIV?

**DOI:** 10.1186/1471-2334-14-S7-O3

**Published:** 2014-10-15

**Authors:** Alina Cibea, Mariana Mărdărescu, Cristina Petre, Ruxandra Drăghicenoiu, Rodica Ungurianu, Sorin Petrea, Ana Maria Tudor, Delia Vlad, Carina Matei, Dan Oțelea, Carmen Crăciun, Tatiana Colțan, Ioana Alina Anca, Mihai Mitran

**Affiliations:** 1 National Institute for Infectious Diseases "Prof. Dr. Matei Balş", Bucharest, Romania; 2 Carol Davila University of Medicine and Pharmacy, Bucharest, Romania; 3 The Institute for Mother and Child Protection “Alfred Rusescu”, Bucharest, Romania; 4 Clinical Hospital of Obstetrics and Gynecology “Prof. Dr. Panait Sârbu”, Bucharest, Romania

## Background

A successful therapeutic course in children infected with HIV relies heavily on their adherence to antiretroviral treatment (ART). The adherence is influenced by every child’s particularities, his family or caregivers and, last but not least, by the selected therapeutic regimen. In newborns and toddlers, who depend completely on their family or caregivers, issues affecting adherence are essential.

## Methods

We analyzed a lot of 43 children infected with HIV through mother to child transmission – newborns and toddlers – born between 2008 and 2013, focusing on specific factors of the caregivers that affected adherence to ART. From the patients’ files we collected data about the prescribed regimen, the last prescription date, the syrup quantity prescribed per day, the number of days prescribed in conjunction with regular assessment of the viral load. Caregivers may include the child’s parents, grandparents, other relatives, or guardians who directly influenced the child’s medication dosing.

## Results

84% of children were born to mothers recently infected with HIV, either through unsafe sex or injecting drug use. Adherence in this group of children maintained a value of 50% compared with the value of 58% in children born to mothers who belong to the 1988-1990s cohort. Assessment of adherence from the viewpoint of socioeconomic status emphasized a 54% value in families with precarious economic status and a 60% value for families with high economic and social levels. Children from mothers with low levels of education were 42% adherent to treatment, compared with 63% adherence in children whose mothers accessed basic or academic education. One of the most significant differences was registered in institutionalized children - 80% compared to children in biological families - 47%.

## Conclusion

Despite of the latest progress in the treatment destined to children with HIV infection – from the discovery of new drugs to formulas tailored to children of various ages (syrup, chewable tablets) – ART options for newborns and toddlers continue to be limited. Given this, adherence to treatment of this pediatric category depends fully on their family or caregivers. In order to make this a reality it is essential to encourage families to access medical services and help them understand the evolution of the diseases and the benefits of treatment.

